# User Experience of Access to Sexual Assault Nurse Examiner and Emergency Contraception in Emergency Departments in the United States: A National Survey

**DOI:** 10.5811/westjem.18405

**Published:** 2024-02-28

**Authors:** Colleen Cowdery, Diana Halloran, Rebecca Henderson, MA Kathleen M. Allen, Kelly O’Shea, Kristen Woodward, Susan Rifai, Scott A. Cohen, Muhammad Abdul Baker Chowdhury, Cristina Zeretzke-Bien, Lauren A. Walter, Marie-Carmelle Elie-Turenne

**Affiliations:** * University of Florida College of Medicine, Gainesville, Florida; † University of Florida College of Public Health and Health Professions, Gainesville, Florida; ‡ Department of Emergency Medicine University of Florida, Gainesville, Florida; § Department of Emergency Medicine, School of Medicine, University of Alabama at Birmingham, Birmingham, Alabama

**Keywords:** emergency contraception, sexual assault nurse examiner, sexual assault

## Abstract

**Background:**

Despite the prevalence of sexual assault presentations to emergency departments (ED) in the United States, current access to sexual assault nurse examiners (SANE) and emergency contraception (EC) in EDs is unknown.

**Methods:**

In this study we employed a “secret shopper,” cross-sectional telephonic survey. A team attempted phone contact with a representative sample of EDs and asked respondents about the availability of SANEs and EC in their ED. Reported availability was correlated with variables including region, urban/rural status, hospital size, faith affiliation, academic affiliation, and existence of legislative requirements to offer EC.

**Results:**

Over a two-month period in 2019, 1,046 calls to hospitals were attempted and 960 were completed (91.7% response rate). Of the 4,360 eligible hospitals listed in a federal database, 960 (22.0%) were contacted. Access to SANEs and EC were reported to be available in 48.9% (95% confidence interval [CI] 45.5–52.0) and 42.5% (95% CI 39.4–45.7) of hospitals, respectively. Access to EC was positively correlated with SANE availability. The EDs reporting SANE and EC availability were more likely to be large, rural, and affiliated with an academic institution. Those reporting access to EC were more likely to be in the Northeast and in states with legislative requirements to offer EC.

**Conclusion:**

Our results suggest that perceived access to sexual assault services and emergency contraception in EDs in the United States remains poor with regional and legislative disparities. Results suggest disparities in perceived access to EC and SANE in the ED, which have implications for improving ED practices regarding care of sexual assault victims.

Population Health Research Capsule What do we already know about this issue?

*In 2005 an estimated 16% of emergency departments (EDs) in the US provided unrestricted access to emergency contraception (EC). Shifting legislation may have impacted access.*

What was the research question?

*What factors affect the user experience of seeking EC and sexual assault nurse examiner (SANE) care in US EDs?*

What was the major finding of the study?

*Access to SANE and EC were reported to be available in 48.9 (95% CI 45.5–52.0) and 42.5% (95% CI 39.4–45.7) of hospitals, respectively.*

How does this improve population health?

*Access to SANE care and EC in US EDs is low and with clear disparities. Results have implications for improving ED policies regarding care of sexual assault victims.*

## INTRODUCTION

The emergency department (ED) is an important point of entry for victims of rape, trafficking, and other forms of sexual and domestic violence. In the United States, sexual assault presentations to EDS increased by 1,533% from 2006 to 2019.^1^ The current state of access to high-quality emergency sexual assault care in the US is unclear.

Sexual assault care in EDs in the US includes the need for forensic evidence collection. A directed approach to provide this specialized care is through the use of sexual assault nurse (or forensic) examiners (SANE)^2^; SANEs are registered nurses or clinicians who have completed a didactic and clinical curriculum approved by the International Association of Forensic Nurses or other certifying body.^3^ They perform forensic sexual assault exams and evidence collection while meeting the medical, psychological, and educational needs of individuals requiring services.^4^ Studies have demonstrated that SANEs provide more “humanizing” care than non-SANE emergency practitioners from the patient perspective,^5^ more comprehensive and consistent medical services,^4^ and more thorough forensic examinations to improve the criminal justice response to sexual assault.^6^ Currently, there are over 450 SANE programs in the US, approximately 75% of which are affiliated with an ED.^3^ ^,^ ^4^ However, no federal regulations dictate who can provide sexual assault care or oversee the quality of care for sexual assault victims, and requirements vary by state.^7^ The state of national access to sexual assault care, including the knowledge of frontline health clinicians about accessibility, is unclear. Despite the effectiveness of SANE-led care,^4^ significant disparities in access are believed to persist.^7^ ^,^ ^8^

In addition to SANE accessibility, emergency contraception (EC) is an important componenent of care after sexual assault, just as it is an important component of reproductive healthcare. Endorsed by leading medical organizations, EC is considered a safe and effective means of preventing pregnancy, including in cases of sexual violence.^9^ ^,^ ^10^ Provision of EC is important in the care of survivors of abuse or domestic violence.^11^Access to EC in the ED is important both as a component of appropriate care for sexual assault and as a service for low-income individuals because cost remains a barrier for them. Indeed, the Affordable Care Act requires most private insurers and state Medicaid programs to cover prescription contraception but not EC.^12^ In 2017, the national average price for trade-name, one-dose levonorgestrel was $49.48 and generic one-dose levonorgestrel was $38.74.^13^ In addition to financial barriers, only 4.9% of pharmacies are open 24 hours per day/seven days per week.^14^ Other potential barriers to patient access include refusal to dispense by pharmacists, misinformation due to personal religious beliefs, lack of clinician exposure, and social stigma.^15^

A 2005 study using a “mystery client” survey found that only an estimated 16% of EDs in the US provide access to EC without restriction.^16^ However, there is reason to believe that access to EC in the ED has changed. The above study was performed prior to notable expansions in EC choices and access in the US. In 2006, the US Food and Drug Administration approved the over-the-counter sale of levonorgestrel to those ≥18 years of age, and then in 2013 expanded access to those ≥15 years.^17^ New hormonal options have also become available.^18^ Further, since 2005 14 states and the District of Columbia have required EDs to dispense EC to sexual assault victims upon request.^19^ Current penalties include fines or suspension or revocation of hospital licensure^20^; however, the absence of strong enforcement mechanisms has correlated with decreased compliance rates.^21^ One 2019 review of literature on EC provision in EDs in the US found that 60% had a policy on EC, 75% officially provided EC counseling, 44% officially offered EC, and 62% officially had EC available to dispense on site.^22^ It is unknown how these statistic correlate with practice.

Most studies have examined access to SANE services and EC in the ED from the perspective of hospital personnel, based on institutional policy, or prior to changes in EC legislation. The studies included only ideal cases rather than real-world conditions; those that used a “mystery client” approach showed lower rates of access.^16^ ^,^ ^22^ Thus, studies conducted from the perspective of the patient or sexual assault victim are needed to define national access and ascertain potential discrepancies between predicted (ie, reported or previously published) and observed rates of access to SANE services and EC in the ED.

Given the recent rise in presentations of sexual assault in the US^1^ and the role of the ED as a pivotal and time-sensitive point of access in cases of sexual violence, we sought to evaluate SANE and EC availability in EDs in the US from the perspective of a patient seeking to know the availability of care over the phone. Our survey addresses user experience, providing a pragmatic example of patient experiences when attempting to access sexual assault services and EC through the ED; this study also examines differences in perceived availability of these services on the basis of geographic and institutional factors.

## MATERIALS AND METHODS

We sought to update the 2005 telephone-based, “secret shopper” study of hospitals across the US to investigate patient access to sexual assault care using the availability of SANE services and EC as a proxy for access to comprehensive services from the perspective of a prospective patient. To assess accessibility and perceived availability, we used the report of frontline healthcare workers likely to be the first point of contact for patients in the ED as the source of information regarding available services. We also sought to determine whether geographic and institutional factors were associated with reported access. Moreover, given the influence of graduate medical education programs on institutional resources, we sought to determine whether teaching status improved access. Our study included a demographic evaluation based on size, rural vs urban setting, teaching status, and faith-based status of hospitals.

We obtained a list of EDs in the US from a publicly available database of the Centers for Medicare & Medicaid Services (CMS) in March 2019. This database consisted of 4,806 hospitals. Exclusion criteria included federal institutions, children’s hospitals, tribal hospitals, hospitals without EDs, and hospitals located in US territories. Of the remaining eligible 4,360 hospitals, 25% were randomly selected and stratified by region (Northeast, South, West, and Midwest; see Table S1 for the list of states per region) and by teaching status. We aimed to survey greater than 20% of eligible hospitals with 21% representation of teaching institutions, which was the proportion of teaching institutions in the overall cohort. Hospitals were classified as teaching hospitals on the basis of their registration with the CMS. Each regional sample was checked to ensure representation of hospitals classified as having teaching status. In general, for every three non-teaching institutions, one teaching institution existed in the analysis within each region.

For the analysis, hospitals were classified by region and state as small (<100 beds), mid-sized (100–200 beds), or large (>200); as urban (population ≥50,000) or rural (population <50,000); as academic or non-academic; as faith- or non-faith-based; and by the presence of a state legislative requirement to offer EC to sexual assault victims. A team of five women investigators simulating potential patients called publicly available ED phone numbers for each hospital between June–September 2019, seeking EC as described by Harrison et al.^16^ Callers contacted the ED seeking medical advice and asked about EC and SANE access. The respondent would either provide the response or transfer the call to a more knowledgeable member of the medical staff including advanced practice providers and physicians.

Callers received structured training with standardized scripts, which were then calibrated through a series of simulated calls. In addition, 5% of calls were screened for fidelity and to ensure standardization by completing a series of observed call encounters. The phone numbers of the callers were concealed, and the time of the day and day of the week was recorded; calls took place during normal business hours (ie, 9 am-5 pm). Callers first asked about access to EC and then asked if it was available in the case of sexual assault. They then asked whether a SANE was available. This script was modeled on the protocol of the most recent survey of EC access.^16^ Following the first 5% of calls, the script was revised and standardized for increased fidelity in data collection. Revisions included minor changes in wording and order of questions.

Primary outcomes were reported access to SANEs and EC in the ED. A SANE was considered available if the respondent reported that a SANE was on site or could be on site within six hours. A SANE was considered not available if respondents were told there were no SANEs available within six hours. We defined EC access as full, conditional, or no access. Full access included hospitals that reported that they had available EC with no restriction. Conditional access was defined as hospitals that reported that they provided EC only in the circumstance of sexual assault; and no access was defined as hospitals that reported an absence of EC provision under any conditions or if the caller was referred to an outpatient pharmacy for access. Secondary outcomes included type of EC options available, alternative methods of obtaining EC, access to referral to alternative healthcare systems, and access to sexual assault resources. As we sought to pragmatically imitate the experience of a prospective patient calling the ED, callers did not ask for the qualifications of respondents, nor did they ask to be transferred to a physician or nurse, although they took such transfers if they were offered. They recorded the first definitive response they received from any staff member.

We managed all study data in Research Electronic Data Capture v 9.11, hosted at the University of Florida. Statistical analyses were performed using SAS v 9.4 (SAS Institute, Inc, Cary, NC). We initially used descriptive statistics, including means, medians, frequencies, and proportions, to examine survey response representation, variable distribution, and missingness where appropriate. We calculated exact confidence intervals (CI) using the Clopper–Pearson method. Unadjusted and adjusted logistic regression models were used to evaluate the relationship between hospital characteristics and outcomes. We performed an unconditional hierarchical logistic regression model, where EDs were nested in respective states, to assess the predicted probability of an ED providing EC for each state. Each state was added as a random effect.

This study received approval for exemption from the University of Florida Institutional Review Board prior to initiation.

## RESULTS

Between July 2–September 5, 2019, callers attempted to call 1,046 hospitals and completed 960 calls (91.7% response rate). Eighty-six of the calls (8.2%) failed due to the following reasons: failure to contact (25, 2.3%); refusal to answer questions (13, 1.2%); hospital closure (20, 1.9%); no ED (7, 0.6%), or another unclassified reason (21, 2%). The Figure illustrates the flow of hospital inclusion or exclusion through the study. Table 1 presents the characteristics of the 960 hospitals that were successfully surveyed. (See Table S2 for the breakdown of number of hospitals by state.) Sexual assault nurse examiners were reported to be available in 48.9% of the 960 hospitals surveyed (Table 2).

**Figure.**
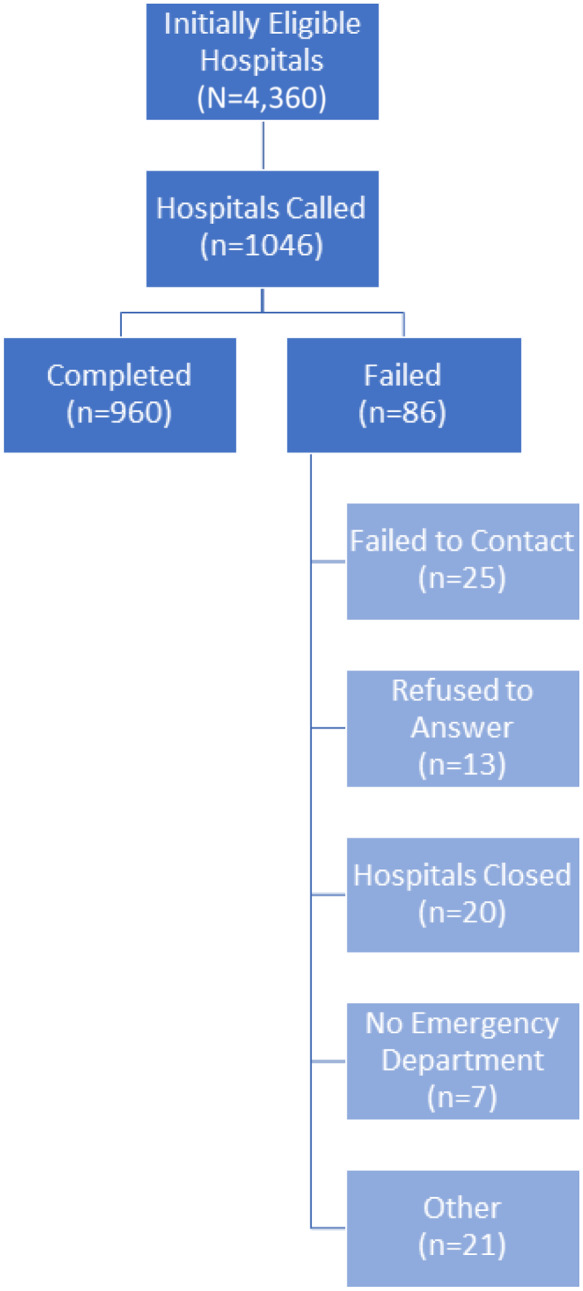
Standards for Reporting of Diagnostic Accuracy diagram reporting flow of participants through the study.

**Table 1.** Hospital characteristics of study sample.

Hospital characteristics	Total (N = 960)	95% CI
Region – n (%)		
Northeast	122 (12.7)	10.7–15.0
Midwest	284 (29.6)	26.7–32.6
South	369 (38.4)	35.4–41.6
West	185 (19.3)	16.8–21.9
Urban–rural status – n (%)		
Urban area	319 (33.2)	30.3–36.3
Rural area	641 (66.8)	63.7–69.8
Size of hospital – n (%)		
Small	469 (48.9)	45.7–52.1
Medium	203 (21.2)	18.6–23.9
Large	288 (30.0)	27.1–33.0
Number of beds – mean ± sd	170.5 ± 206.3	157.4–183.5
Faith-based status – n (%)		
Faith based	173 (18.0)	15.6–20.6
Non-faith based	787 (82.0)	79.4–84.4
Hospital type – n (%)		
Academic	237 (24.7)	22.0–27.5
Non-academic	723 (75.3)	72.5–78.0
State requirement if SA – n (%)		
In-state requiring dispense	284 (29.6)	26.7–32.6
Not required to dispense	612 (63.8)	60.6–66.8
No state law (Ohio and Pennsylvania)	64 (6.7)	5.2–8.4

*CI*, confidence interval; *SA*, sexual assault.

**Table 2.** Emercency contraception survey response by hospital sample.

Survey responses (N = 960)	Frequency (%)	95% CI
EC Access		
Full access^*^	215 (22.4)	19.8–25.2
No access	551 (57.4)	54.2–60.5
Conditional access^**^	193 (20.1)	17.6–22.8
Contraception options (if available, n = 408)		
Levonorgestrel (Plan B)	196 (48.0)	43.1–53.0
Ulipristal (Ella)	6 (1.5)	0.5–3.2
IUD	0 (0.0)	0.0–0.0
Don’t know	216 (52.9)	48.0–57.9
Method of obtaining EC (if available, n = 408)		
Physician decision	256 (62.7)	57.9–67.5
Pregnancy test	12 (2.9)	1.5–5.1
Pelvic exam	26 (6.4)	4.2–9.2
Don’t know	87 (21.3)	17.4–25.6
Other	62 (15.2)	11.9–19.1
Access to referrals (if EC not available, n = 551)		
Yes	341 (61.9)	57.7–66.0
No	207 (37.6)	33.5–41.8
Access to sexual assault resources		
Yes	653 (68.0)	65.0–71.0
No	281 (29.3)	26.4–32.3
Don’t know	23 (2.4)	1.5–3.6
Access to SANEs		
Yes	468 (48.9)	45.5–52.0
No	458 (47.8)	44.5–50.9
Don’t know	32 (3.3)	2.3–4.7

* Full access values are hospitals that answered yes to having EC when initially asked.

** Conditional access values are hospitals that responded no to having EC available initially, but yes when sexual assault was reported.

*CI*, confidence interval; *EC*, emergency contraception; *IUD*, intrauterine device; *SANE*, sexual assault nurse examiner.

After adjusting for covariates, the following factors were independent predictors of SANE access: region; EC access; size of hospital; academic status; and urban status (Table 3). See unadjusted comparisions in Table S2). Region was associated with reported SANE access, with hospitals in the Northeast being 4.00 times more likely (95% CI 2.38–7.14), 2.78 times more likel (95% CI 1.59–4.76), and 2.00 times more likely (95% CI 1.19,−3.33) to have SANE access than hospitals in the South, West, and Midwest, respectively (Table 3). Reported EC access in cases of sexual assault was also associated with SANE presence, with employees at these hospitals 3.94 times more likely (95% CI 2.66–5.83) to report having SANEs when compared to those at hospitals without reported EC access (Table 3).

**Table 3.** Unadjusted and adjusted odds ratios of access to a sexual assault nurse examiner by hospital characteristics (available vs not available).

Hospital characteristics	Unadjusted model OR (95% CI)	Adjusted model OR (95% CI)
Region		
Northeast	Ref	Ref
Midwest	0.43 (0.27–0.69)	0.50 (0.30–0.84)
South	0.26 (0.16–0.40)	0.25 (0.14–0.42)
West	0.35 (0.21–0.57)	0.36 (0.21–0.63)
Urban–rural status		
Urban area	Ref	Ref
Rural area	0.72 (0.55–0.95)	1.48 (1.00–2.20)
Size of hospital		
Small	Ref	Ref
Medium	2.26 (1.61–3.19)	2.96 (1.72–5.11)
Large	2.76 (2.03–3.76)	2.43 (1.63–3.61)
Number of beds (per 250 increase)	1.49 (1.24–1.80)	0.91 (0.70–1.18)
Faith-based status		
Non-faith based	Ref	Ref
Faith based	1.16 (0.83–1.63)	1.01 (0.70–1.46)
Hospital Type		
Non-academic	Ref	Ref
Academic	2.87 (2.08–3.96)	2.18 (1.42–3.34)
State requirement if SA		
In-state requiring dispense	Ref	Ref
Not required to dispense	0.65 (0.49–0.87)	0.95 (0.66–1.38)
No state law (Ohio and Pennsylvania)	1.42 (0.81–2.52)	0.81 (0.42–1.53)
EC access	
No access	Ref	Ref
Full access*	2.87 (2.05–4.00)	2.33 (1.62–3.34)
Conditional access**	4.82 (3.33–6.97)	3.94 (2.66–5.83)

*OR*, odds ratio; *CI*, confidence interval; *SA*, sexual assault; *EC*, emergency conception.

Mid-sized and large hospitals were 2.96 (95% CI 1.72–5.11) and 2.43 (95% CI 1.63–3.61) times more likely, respectively, to report having SANE access than small hospitals. Prior to adjusting for covariates, it appeared as though urban hospitals were more likely to report having SANE access (1.39 times more likely, Table 3). In the adjusted model, however, rural hospitals were 1.48 times more likely (95% CI 1.00–2.20) to report having SANEs, illustrating a reversal of the association with hospital size acting as the qualitative confounder (Table 3). Faith-based and non-faith-based hospitals reported having SANEs available at similar rates of 51.7% and 48.2%, respectively (Table 3). Academic hospitals were 2.18 times per likely (95% CI 1.42–3.34) to report having SANE access than non-academic hospitals (Table 3).

Of the 960 hospitals included, 551 (57.4%) reported no access to EC. Of the 408 (42.5%) reporting EC access, 215 (22.4%) had full access, and 193 (20.1%) had conditional access (Table 2). Of the 551 hospitals with no access, 341 (61.9%) had a referral system to obtain EC. Of the 408 hospitals with reported EC access, 196 (48.0%) prescribed levonorgestrel, six (1.5%) prescribed ulipristal acetate, and 216 (52.9%) of respondents did not know the available options. No hospitals reported the copper intrauterine device (IUD) as an option. The majority of respondents told callers that EDs leave EC provision to the discretion of the physician (62.7%), 2.9% require a pregnancy test, and 6.4% require a pelvic examination (Table 2). Nationally, the predicted probability of a respondent reporting that their hospital did not provide any EC in the ED was 55.2% (Table S3).

Massachusetts, Oregon, New Jersey, New York, Washington, and Wisconsin had a significantly greater predicted probability of reported EC access in EDs than the national average, while Florida, California, Kansas, Louisiana, Texas, and Nebraska had a significantly lower chance of having EC than the national average (Table S3). The presence of a state requirement to prescribe EC for sexual assault victims was the second strongest predictor of EC access (following region), at 2.27 times more likely (95% CI 1.59–3.22). Additionally, rural hospitals were 1.65 times more likely (95% CI 1.11–2.44) than urban hospitals to have any EC access, and academic hospitals were 1.58 times more likely (95% CI 1.05–2.39) t than non-academic hospitals to have any EC access (Table 4; see unadjusted comparisions in Table S4).

**Table 4.** Unadjusted and adjusted odds ratios of emergency contraception access by hospital characteristics (any access* vs no access).

Hospital characteristics	Unadjusted models OR (95% CI)	Adjusted model OR (95% CI)
Region		
Northeast	Ref	Ref
Midwest	0.31 (0.20–0.50)	0.39 (0.24–0.65)
South	0.16 (0.10–0.26)	0.25 (0.15–0.43)
West	0.32 (0.20–0.53)	0.33 (0.19–0.57)
Urban–rural status		
Urban area	Ref	Ref
Rural area	1.05 (0.80–1.37)	1.65 (1.11–2.44)
Size of hospital		
Small	Ref	Ref
Medium	1.31 (0.94–1.84)	1.35 (0.91–2.00)
Large	1.59 (1.81–2.15)	1.60 (0.93–2.73)
Number of beds (per 250 increase)	1.18 (1.01–1.39)	1.07 (0.83–1.38)
Faith-based status		
Non-faith based	Ref	Ref
Faith based	0.91 (0.65–1.28)	0.90 (0.62–1.30)
Hospital type		
Non-academic	Ref	Ref
Academic	1.67 (1.25–2.25)	1.58 (1.05–2.39)
State requirement if SA		
In-state requiring dispense	Ref	Ref
Not required to dispense	0.33 (0.25–0.45)	0.44 (0.31–0.63)
No state law (Ohio and Pennsylvania)	0.93 (0.54–1.62)	0.55 (0.30–1.02)

Any access* includes hospitals with full access** or conditional access***.

Full access** values are hospitals that answered yes to having emergency contraception available when initially asked.

Conditional access*** values are hospitals that responded no to having EC available initially, but yes when sexual assault was reported.

*OR*, odds ratio; *CI*, confidence interval; *SA*, sexual assault.

After adjusting for covariates, reported EC access was associated with hospital region, urban status, academic status, and state requirement in cases of sexual assault (Table 4). After excluding hospitals with reported conditional access in cases of sexual assault, faith-based status became an additional independent predictor, while the association between academic status and EC access was no longer significant (Table 5).

**Table 5.** Unadjusted and adjusted odds ratios of full emergency contraception access by hospital characteristics (full access* vs no access).

Hospital characteristics	Unadjusted models OR (95% CI)	Adjusted model OR (95% CI)
Region		
Northeast	Ref	Ref
Midwest	0.22 (0.13–0.37)	0.32 (0.18–0.59)
South	0.14 (0.08–0.23)	0.23 (0.12–0.45)
West	0.30 (0.18–0.54)	0.33 (0.18–0.63)
Urban–rural status		
Urban area	Ref	Ref
Rural area	1.14 (0.81–1.60)	1.74 (1.05–2.87)
Size of hospital		
Small	Ref	Ref
Medium	1.34 (0.75–1.72)	1.11 (0.67–1.82)
Large	1.42 (0.99–2.04)	1.28 (0.67–2.46)
Number of beds (per 250 increase)	1.16 (0.97–1.39)	1.16 (0.86–1.55)
Faith-based status		
Non-faith based	Ref	Ref
Faith based	0.45 (0.27–0.75)	0.44 (0.25–0.76)
Hospital type		
Non-academic	Ref	Ref
Academic	1.61 (1.12–2.30)	1.67 (0.99–2.82)
State requirement if SA		
In-state requiring dispense	Ref	Ref
Not required to dispense	0.32 (0.22–0.45)	0.42 (0.27–0.67)
No state law (Ohio and Pennsylvania)	0.93 (0.54–1.62)	0.54 (0.25–1.14)

Full access* values are hospitals that answered yes to having emergency contraception when initially asked.

*OR*, odds ratio; *CI*, confidence interval; *SA*, sexual assault.

## DISCUSSION

Globally, rates of sexual assault, gender-based violence, and human trafficking for sexual exploitation remain high, and access to appropriate care following a sexual assault remains marked by sharp disparities.^23^ ^–^ ^25^ Similarly, our study suggests that there is inconsistent access to SANEs and EC in EDs across the US. While this study does not establish the distribution of absolute access, our methodology provides a pragmatic depiction of the patient experience when attempting to access sexual-assault services and EC through an ED. This picture reflects stark disparities in access as well as overall low levels of access to SANEs and EC nationally. Our findings highlight the difference between policy and practice, which may be influenced by bias, lack of knowledge of policy by clinicians, and other factors.

Roughly half of the EDs surveyed reported that they could not provide SANEs for sexual assault victims on site within six hours, and responders in the South were twice as likely not to know whether there was a SANE available. This finding is in contrast to other studies conducted in the Southeastern US that relied on clinician and administrator surveys, which found that access to SANE and EC was consistent with the standard of care.^26^ It is, therefore, unclear whether this regional difference represents true availability or a gap in the education of frontline emergency clinicians in the southern US.

Larger academic institutions were more likely to have a SANE available, possibly because for those institutions it was less of a financial burden. The cost to develop a SANE program can be up to $40,000.^27^ According to the International Association of Forensic Nursing (IAFN), only 1,200 IAFN-certified SANEs for adults and adolescents are available internationally.^28^ As a result, disparities in access are likely, and although the reasons are not well studied, they likely include a number of variables such as high costs, limited training opportunities, and a lack of supportive resources, particularly in already underserved areas.^29^ Our results, in combination with the increase in the number of sexual assault patients being seen in the ED,^1^ highlight the need for hospitals to be prepared with properly trained staff to treat this patient population. One possible solution to the cost of SANE services for individual hospitals is to regionalize resources.

In the unadjusted model, rural hospitals appeared less likely to have SANEs available; however, once adjusted for hospital size, rural hospitals were more likely to report having a SANE. This is contrary to previous research in Pennsylvania, Washington, and Oregon, which demonstrated that programs in rural areas were lacking in SANEs and facilities, resulting in urban programs absorbing patients from underserved areas,^7^ ^,^ ^8^ This may be a result of the availability of sexual assault resources outside the ED in urban areas, or of the centralization of SANEs at a single hospital in an urban center. If the results of this study represent access to SANEs, rather than a lack of knowledge among frontline healthcare practitioners, there is a strong disparity in SANE access for sexual assault patients based on region and hospital size. This disparity may affect the quality of counseling and forensic evidence collection based on the location of the hospital, which could have legal ramifications for victims as hospitals in different locations may not equally facilitate the collection of high-quality evidence in cases of sexual assault.

Only 22.4% of ED frontline healthcare practitioners reported that they provide EC without restriction; furthermore, an additional 20.1% reported that they provided EC only in cases of sexual assault. Our results align with those reported by Harrison et al in 2005, with a minority (31.5%) of surveyed EDs found to provide EC.^16^ The poor access to EC found in this study may in part reflect increased access to alternative resources, such as over-the-counter EC at pharmacies or women’s specialty clinics. The low rate of access reported by ED personnel may also be due to lack of knowledge of hospital policies regarding EC among frontline ED staff, especially about costs and barriers associated with these alternative resources.^27^ Similar to what Harrison et al reported, respondents in our study frequently provided incorrect or misguided comments regarding EC. Several respondents referred to EC as an “abortion pill,” possibly mifepristone, or a hysterectomy during the phone call. According to the ED non-physician practitioners surveyed, 63% of EC provision was based on individual physician discretion, which is not required in many states.

Studies have demonstrated that less than 50% of victims of sexual assault seek medical attention. While the reasons are multifactorial, it is clear that victims experience serious psychosocial and emotional stress that may contribute to a reluctance to be subject to additional scrutiny, loss of privacy, or invasive examinations.^30^ In our survey, many reported that EC was dispensed only following a physician assessment, which would include a pelvic examination. Many respondents in our study stated that their ED did not take sexual assault cases and that the patient would need to be transferred to another facility or seek guidance from law enforcement.

Few respondents provided the specific brand of EC available, and none offered the copper IUD as an option. Many respondents commonly referred patients to private pharmacies for EC, a problematic practice given coverage of costs and potential logistical difficulties and delays. Importantly, the referral of those seeking EC to private pharmacies limits access to consultation on sexually transmitted disease, behavioral health, or the opportunity to report to law enforcement in the case of victims of sexual assault, domestic violence, or trafficking. These findings underscore the need for increased training for healthcare practitioners responsible for triage and response to inquiries.

In states with legislation requiring access to EC in cases of sexual assault, EDs were more than twice as likely to report that EC was available without restriction, demonstrating that such legislation may have an impact. With current enforcement mechanisms in place for only 13 states, there is room for expansion of legislation to cover the remaining states.

Perhaps unexpectedly, EC was more likely to be available in rural hospitals after adjusting for covariates. Rural hospitals often serve as critical access points for remote or underserved communities. Non-faith-based hospitals were more than twice as likely to report providing EC than faith-based hospitals, consistent with a previous study in which non-Catholic hospitals were more likely to provide EC than Catholic hospitals.^21^ This finding may be based on local institutional policies limiting access among faith-based institutions.

There is a need for improved education on sexual assault care, as well as an increase in SANE access among hospitals. Hospitals should consider building SANE resources into ED protocols. Hospital administrators can collaborate with local rape crisis centers or apply for federal grants or funding to defray the cost of training and supplies.

## LIMITATIONS

The primary limitation of this study was the inconsistency in knowledge of protocols related to this topic and willingness to provide accurate information over the telephone. It is plausible that callers would have received different information had the encounter been in person. However, a phone protocol was specifically chosen as a pragmatic approach used by a potential member of the community seeking services.

The specific inquiry regarding sexual assault rather than the initial request for EC may have influenced the respondent’s response regarding resources and access. Respondents in this study may have been more motivated to find an answer to questions when the topic of sexual assault was introduced. For example, some respondents who stated EC was not available changed their response upon the callers’ disclosure that there had been a sexual assault. When respondents endorsed SANE access, callers did not record on-site availability, nor the hours when access was available. Call timing was varied randomly between 9 am–5 pm but was not standardized. In addition, as many sexual assaults present outside normal working hours, it is possible that the availability could be even lower during off-hours.

## CONCLUSION

Access to emergency contraception and sexual assault nurse examiners in EDs remains limited with disparities in access across the nation. Variable accessibility depending on the geographic location of the hospital or the legislative status of the state suggests that those seeking these resources might receive substandard quality of healthcare depending on the institution where they have chosen to seek care. Given the importance of EC and sexual assault services, emergency physicians may find it worthwhile to examine their hospitals’ existing protocols regarding dispensing prescriptions of these medications and availability of SANEs. Hospitals should consider providing training for all ED staff, especially those who first interact with patients, to prevent misinformation about patient access to EC or SANEs.

## Supplementary Information




