# Supplementary material for: User Experience of Access to Sexual Assault Nurse Examiner and Emergency Contraception in Emergency Departments in the United States: A National Survey
Source: West J Emerg Med. 2024 Feb 28;25(2):291–300. doi: 10.5811/westjem.18405 (PMC11000551; doi:10.5811/westjem.18405)
Supplement: Supplementary file 1 [file wjem-25-291-s001.docx]

**Table S1.** States by region.

| Northeast | South | Midwest | | West |
| --- | --- | --- | --- | --- |
| Connecticut | Alabama | Illinois | | Alaska |
| Maine | Arkansas | Indiana | | Arizona |
| Massachusetts | Delaware | Iowa | | California |
| New Hampshire | Florida | Kansas | | Colorado |
| New Jersey | Georgia | Michigan | | Hawaii |
| New York | Kentucky | Minnesota | | Idaho |
| Pennsylvania | Louisiana | Missouri | | Nevada |
| Rhode Island | Maryland | Montana | | New Mexico |
| Vermont | Mississippi | Nebraska | | Oregon |
|  | North Carolina | North Dakota | | Utah |
|  | Oklahoma | Ohio | | Washington |
|  | South Carolina | South Dakota | | Wyoming |
|  | Tennessee | Wisconsin | |  |
|  | Texas |  | |  |
|  | Virginia | |  |  |
|  | West Virginia | |  |  |

**Table S2.** Unadjusted comparisons between hospital characteristics and availability of sexual assault nurse
examiner* (3-group comparison).

|  | Available  (n=468) | Not available  (n=458) | Unknown  (n=32) | *P*-value |
| --- | --- | --- | --- | --- |
| Region – n (%) |  |  |  | <0.001 |
| Northeast | 87 (71.3) | 32 (26.2) | 3 (2.5) |  |
| Midwest | 150 (52.8) | 127 (44.7) | 7 (2.5) |  |
| South | 143 (39.0) | 206 (56.1) | 18 (4.9) |  |
| West | 88 (47.6) | 93 (50.3) | 4 (2.2) |  |
| Urban–rural status – n (%) |  |  |  | 0.042 |
| Urban area | 171 (53.8) | 134 (42.1) | 13 (4.1) |  |
| Rural area | 297 (46.4) | 324 (50.6) | 19 (3.0) |  |
| Size of hospital – n (%) |  |  |  | <0.001 |
| Small | 178 (38.0) | 279 (59.5) | 12 (2.6) |  |
| Medium | 144 (56.2) | 79 (38.9) | 10 (4.9) |  |
| Large | 176 (61.5) | 100 (35.0) | 10 (3.5) |  |
| Number of beds – mean ± SD | 199.0 ± 195.3 | 139.6 ± 216.2 | 178.6 ± 141.3 | <0.001 |
| Faith-based status – n (%) |  |  |  | 0.677 |
| Faith based | 89 (51.7) | 77 (44.8) | 6 (3.5) |  |
| Non-faith based | 379 (48.2) | 381 (48.5) | 26 (3.3) |  |
| Hospital type – n (%) |  |  |  | <0.001 |
| Academic | 156 (66.1) | 68 (28.8) | 12 (5.1) |  |
| Non-academic | 312 (43.2) | 390 (54.0) | 20 (2.8) |  |
| State requirement if SA – n (%) |  |  |  | 0.003 |
| In-state requiring dispense | 157 (55.3) | 120 (42.3) | 7 (2.5) |  |
| Not required to dispense | 270 (44.3) | 316 (51.8) | 24 (3.9) |  |
| Other* (Ohio and Pennsylvania) | 41 (64.1) | 22 (34.4) | 1 (1.6) |  |

**Table S3.** Predicted probability of NOT having any emergency conception availability, by state.

| State | Probability (%) | *P*-value | State | Probability (%) | *P*-value |
| --- | --- | --- | --- | --- | --- |
| Alabama | 77.2 | 0.063 | Montana | 58.8 | 0.751 |
| Alaska | 47.3 | 0.629 | **Nebraska** | **82.0** | **0.026** |
| Arizona | 51.9 | 0.819 | Nevada | 81.4 | 0.063 |
| Arkansas | 71.6 | 0.139 | New Hampshire | 44.0 | 0.437 |
| **California** | **73.5** | **0.020** | **New Jersey** | **27.9** | **0.036** |
| Colorado | 59.8 | 0.701 | New Mexico | 69.2 | 0.260 |
| Connecticut | 31.1 | 0.112 | **New York** | **37.1** | **0.045** |
| Delaware | 46.7 | 0.639 | North Carolina | 36.0 | 0.063 |
| **Florida** | **78.3** | **0.002** | North Dakota | 65.1 | 0.449 |
| Georgia | 62.6 | 0.444 | Ohio | 49.3 | 0.513 |
| Hawaii | 61.5 | 0.676 | Oklahoma | 71.0 | 0.131 |
| Idaho | 27.5 | 0.097 | **Oregon** | **29.3** | **0.022** |
| Illinois | 45.2 | 0.265 | Pennsylvania | 38.7 | 0.065 |
| Indiana | 63.1 | 0.449 | Rhode Island | 46.7 | 0.639 |
| Iowa | 47.8 | 0.420 | South Carolina | 33.7 | 0.126 |
| **Kansas** | **79.5** | **0.012** | South Dakota | 48.8 | 0.568 |
| Kentucky | 51.0 | 0.690 | Tennessee | 68.5 | 0.219 |
| **Louisiana** | **80.6** | **0.007** | **Texas** | **77.9** | **0.001** |
| Maine | 29.6 | 0.054 | Utah | 58.4 | 0.804 |
| Maryland | 60.8 | 0.618 | Vermont | 39.3 | 0.289 |
| **Massachusetts** | **20.4** | **0.017** | Virginia | 61.5 | 0.549 |
| Michigan | 63.5 | 0.404 | **Washington** | **28.7** | **0.005** |
| Minnesota | 38.1 | 0.061 | West Virginia | 62.6 | 0.583 |
| Mississippi | 73.8 | 0.081 | **Wisconsin** | **34.4** | **0.022** |
| Missouri | 73.8 | 0.081 | Wyoming | 55.4 | 0.985 |

*Note:* National predicted probability of not having any emergency conception among hospital populations = 55.2% (95% confidence interval 47.4–62.7%).

**Bolded states** have a significantly different probability than the national average.

**Table S4.** Unadjusted comparisons between hospital characteristics and EC availability (3-group comparison).

| Hospital characteristics | Full availability | Conditional availability | No availability | *P*-value |
| --- | --- | --- | --- | --- |
| Region – n (%) |  |  |  | 0.003 |
| Northeast | 54 (44.3) | 34 (27.9) | 34 (27.9) |  |
| Midwest | 55 (19.4) | 72 (25.4) | 157 (55.3) |  |
| South | 57 (15.5) | 52 (14.1) | 259 (70.4) |  |
| West | 49 (26.5) | 35 (18.9) | 101 (54.6) |  |
| Urban–rural status – n (%) |  |  |  | 0.657 |
| Urban area | 66 (20.8) | 67 (21.1) | 185 (58.2) |  |
| Rural area | 149 (23.2) | 126 (19.7) | 366 (57.1) |  |
| Size of hospital – n (%) |  |  |  | 0.021 |
| Small | 100 (21.3) | 77 (16.4) | 292 (62.3) |  |
| Medium | 44 (21.7) | 46 (22.7) | 113 (55.7) |  |
| Large | 71 (24.7) | 70 (24.4) | 146 (50.9) |  |
| Number of beds – mean ± SD | 187.6 ± 222.2 | 184.6 ± 170.3 | 158.2 ± 210.2 | 0.002 |
| Faith-based status – n (%) |  |  |  | <0.001 |
| Faith based | 20 (11.6) | 50 (29.1) | 102 (59.3) |  |
| Non-faith based | 195 (24.8) | 143 (18.2) | 449 (57.1) |  |
| Hospital type – n (%) |  |  |  | 0.003 |
| Academic | 63 (29.7) | 60 (25.4) | 113 (47.9) |  |
| Non-academic | 152 (21.0) | 133 (18.4) | 438 (60.6) |  |
| State requirement if SA – n (%) |  |  |  | <0.001 |
| In-state requiring dispense | 92 (32.4) | 77 (27.1) | 115 (40.5) |  |
| Not required to dispense | 104 (17.0) | 98 (16.0) | 409 (66.9) |  |
| Other (Ohio and Pennsylvania) | 19 (29.7) | 18 (28.1) | 27 (42.2) |  |

*SA*, sexual assault.
